# The paradigm shift towards online learning during Covid-19 pandemic: an assessment of the attitudes on the learning practices among University of Zambia pharmacy students

**DOI:** 10.1186/s12909-023-04433-8

**Published:** 2023-06-20

**Authors:** Martin Kampamba, Kaluba Chiluba, Christabel Nang’andu Hikaambo, Enala S. Lufungulo, Kennedy Mwila

**Affiliations:** 1 Department of Pharmacy, School of Health Sciences, University of Zambia, Lusaka, Zambia; 2 Graduate School of Education, Peking University, Beijing, China

**Keywords:** Online learning, COVID-19, Pharmacy students, Attitudes, Learning practices

## Abstract

**Background:**

The nexus between higher education and digital technology has been extensively studied in the past and recently during the COVID-19 pandemic. This study aims to ascertain pharmacy students' attitudes towards using online learning during COVID-19.

**Methods:**

This cross-sectional study assessed the University of Zambia’s (UNZA) pharmacy students’ adaptive characteristics, such as attitude, perception, and barriers to online learning during the COVID-19 pandemic. Data from a survey of *N* = 240 were collected using a self-administered, validated questionnaire along with a standard tool. Findings were statistically analysed using STATA version 15.1.

**Results:**

Of the 240 respondents, 150 (62%) had a negative attitude towards online learning. Further, 141 (58.3%) of the respondents find online learning less effective than traditional face-to-face learning. Regardless, 142 (58.6%) of the respondents expressed a desire to modify and adapt online learning. The mean scores for the six domains of attitude (perceived usefulness, intention to adapt, ease of use of online learning, technical assistance, learning stressors, and distant use of online learning) were 2.9, 2.8, 2.5, 2.9, 2.9, and 3.5, respectively. After multivariate logistic regression analysis, no factors in this study were significantly associated with attitude towards online learning. The high cost of the internet, unreliable internet connectivity and lack of institutional support were perceived barriers to effective online learning.

**Conclusion:**

Although most of the students in this study had a negative attitude toward online learning, they are willing to adopt it. Online learning could supplement traditional face-to-face learning in pharmacy programs if it can be made more user-friendly, have fewer technological barriers, and be complemented by programs that help improve practical learning abilities.

## Background

It is impossible to deny the extent of information technology's influence and impact on many facets of our lives today, as well as its rising popularity and use in the educational field [1]. The ongoing COVID-19 pandemic, which at one point forced all educational institutions worldwide to close its doors, has made this function in the academic sector more crucial. This has led to a number of difficulties at all levels of education especially for students [2].

This pandemic threatens human health, social and political patterns. It significantly impacts educational systems, which have negative feedback that inhibits the social, economic, and political well-being of two communities, societies, and nations [3]. The COVID-19 pandemic has affected educational systems worldwide, leading to the widespread closure of schools in the affected countries. Over 1.7 billion students were out of school as of March 28, 2020, due to schools being closed due to the pandemic [4].

Lockdowns in response to COVID-19 have disrupted traditional education in the majority of countries by forcing widespread school closure [5]. Despite the efforts of the educational community to maintain learning continuity during this time, children and students have had to rely more on their own resources, such as the internet, television, and radio, to continue studying remotely. Teachers have also had to adapt to new adult educational concepts and teaching styles [6].

The upsurge in knowledge growth compels online learning via computer networks, provides educational content, and enables interaction between the students and the teacher. As a result, academic institutions around the world strive to make the most of technology advancements in order to improve the teaching and learning experience for a variety of students, faculty members, and administrators [7]. Online learning refers virtual learning environment with no physical peers. However there is flexibility in time and space exist, allowing for flexible learning, and it offers an alternative for people who cannot attend face-to-face classrooms [8]. Both students and lecturers consider online technology a helpful part of our learning system. As Habes M noted, better communication technologies generally facilitated learning systems, as accessibility to social media is a useful, valuable source of information and communication. Many nations provided television broadcasts in addition to online resources to facilitate distant learning throughout the pandemic [9].

The role of attitudes in comprehending how learners use new learning environments cannot be overstated because attitudes are regarded as predictors of behavior [10]. Experts have found that attitude to some extent predicts whether someone would engage in a particular behaviour. Therefore, students that have a positive attitude are therefore more likely to accept an e-learning system [11]. Attitude is influenced by individual’s sociocultural upbringing, previous knowledge, and prior learning experiences [12]. According to some studies, previous online learning experience, access to home internet, studying in technical field and owning a computer are some of factors that have been found to influence positive attitude towards online learning [13]. The benefits and drawbacks that students feel from using online learning also have an impact on their attitudes toward it. Their attitude will therefore either be positive if the online learning system suits their needs or negative if they are unable to adjust to it because they lack the necessary attributes [14]. It is clear that adopting a positive attitude toward onlearning is essential to ensuring that students receive the full educational benefits from this delivery method [15].

Compared to traditional face-to-face learning, obstacles such as a lack of skills, barriers, and access to Information and Communication Technology (ICT) can cause negative attitudes and perceptions towards online learning [16]. Therefore, this study aimed at assessing pharmacy students’ adaptive characteristics, such as attitude, perception, and barriers to online learning during the COVID-19 pandemic.

## Method

### Study design and setting

This study used a cross-sectional design with both an online and a physical survey with voluntary participants from pharmacy students at the University of Zambia to investigate pharmacy students' perceptions and attitudes toward online learning during COVID-19. The research was carried out over four weeks in August 2022 at the University of Zambia's Ridgeway campus in Lusaka. We used an online and physical survey because some classes were in session while others were on a short break.

### Study population

The population of this research study was all the students enrolled in the pharmacy program, which is 600.

### Sample size determination and sampling technique

Taro Yamane’s formula was adopted to determine the sample size for this study. 

n = N/1 + Ne^2^, where n = Sample size desired, N = Total population, and e = Level of precision, margin, or sampling error (assumed as 0.05 [= ± 5%]) at a 95% confidence level, *n* = 600/[1 + (600 X 0.05^2^)], n = 240 students

A complete class list of registered pharmacy students (first, second, third, fourth, and fifth-year levels) was compiled for the 2021–2022 academic year. Each list element was given a random number using a table of random digits. The list was then sorted in ascending order, and the first n elements were chosen to participate in the survey, where n is equal to the sample size. The proportion to size assumption was used to split the sample size among all classes as follows: 5^th^ year = (130/600) × 240 = 52 students, then in 4^th^ year = (102/600) × 240 = 41 students, then also in 3^rd^ year = (178/600) × 240 = 71 students, then in 2^nd^ year = (193/600) × 240 = 77 students.

### Data collection

Data collection was achieved through the questionnaire's online and physical distribution. The online questionnaire was prepared in Google Forms, and the link was shared with selected pharmacy students. The hard copy questionnaire was physically distributed to the selected participants.

The questionnaire was divided into four components and was modified with the authors' permission from previously published study [17]. Part 1: Questionnaire on socio-demographic data (gender, age, marital status, residence, year of study, online learning devices utilised, online resources used, and prior experience). Part 2. Consisted of a Likert scale (scale 1 = strongly effective to 5 = strongly ineffective) comparing the effectiveness of online learning against traditional face-to-face learning. Part 3 of the standard Likert scale measured how students felt about online learning. The questionnaire measured attitudes about online learning and had six domains that made up the scale: perceived usefulness (from 1 to 18), intention to adapt to e-learning (19 to 27), ease of e-learning use (28 to 35), technical support (36 to 39), stresses associated with e-learning (40 to 42), and remote use of online learning (43 to 46). The scale ran from 1 to 5, with 1 being “strongly disagree” and 5 being “strongly agree.” There were 46 items in all six domains, with 26 being positively and 20 being negatively worded. The range of the overall score was 46 to 230. Part 4 related to barriers towards online learning during the Covid -19 pandemic.

The adopted instrument's validity was examined through literature research and consultation with subject specialists. Pre-testing the instrument with 10% (24 pharmacy students) of an estimated sample population of a similar kind in a comparable situation allowed researchers to assess the instrument's reliability and internal consistency. In this study, the reliability Cronbach’s alpha score on the Likert scale for the six domains was 0.83 for perceived usefulness, 0.78 for intention to adapt, 0.76 for ease of online learning, 0.81 for technical support, 0.66 for online learning stressors, and 0.79 for distant use of online learning.

The reliability of Cronbach’s alpha score on the Likert scale for the effectiveness of online learning against traditional face-to-face learning and barriers to online learning was 0.67 and 0.81, respectively. Cronbach's alpha for the instrument's overall reliability was 0.91, indicating acceptable internal consistency.

### Ethical considerations

Ethical guidelines serve as a basis for reliable, legitimate, and representative principles, rules, norms, designs, and conduct [18]. Ethical values were considered in this study by obtaining a written consent letter from the University of Zambia Ridgeway Campus Research Committee (UNZAHSREC) [Protocol ID number: 202211231162]. Participation was voluntary, so participants were not forced to participate in the study. Additionally, written consent was also obtained from the participants. The study posed no risks to the participants and provided no direct benefit to them. The purpose of the study was explained to the participants, and they were free to skip any questions they were not familiar with, or that might stress them out. Utmost confidentiality and anonymity were maintained because no names appeared on the questionnaires. No compensation was given to any participant for participating in the study, and the information provided was solely for research purposes.

### Data analysis

The statistical data analysis was conducted using STATA version 15.1, and Microsoft Excel 2016 was used to generate the figures. The data were analysed and interpreted using inferential statistics and descriptive statistics (frequency, mean, percentage, and standard deviation). Overall attitude was dichotomised as negative and positive based on the mean score of the 5-point Likert scale. Bivariate logistic regression analysis was performed to determine the association of each independent variable with attitude toward online learning. The independent variables with a *p*-value < 0.2 in the bivariate analysis were included in the multivariable logistic regression model to identify predictors of attitude toward online learning. A *p*-value of < 0.05 was considered statistically significant in all analyses.

## Results

### Socio-demographic

Table 1 shows the socio-demographic data of the respondents. More than half of the participants in this study, 138 (57.0%), were male, and the majority, 188 (77.7%), were between the ages of 18 and 24 years, with the majority, 232 (95.9%), being single. The vast majority of 215 (88.8%) respondents were unemployed, and most of them came from 190 (78.5%) urban areas. It was found that 210 (86.8%) of the participants used smartphones for their online learning, and 205 (84.7%) used mobile data as a source for the internet. 235 (97.1%) of the participants (2^nd^ to 5^th^ year) had participated in online learning during this pandemic, but the majority 81 (33.5%) were in the 2^nd^ year of study.

**Table 1:** Socio-demographic characteristics of the respondents (*n* = 242)

Variables	Category	Frequency	Percentage
Gender	Male	138	57.0
	Female	104	43.0
Age	18–24	188	77.7
	25–29	41	16.9
	< 30	13	5.4
Marital status	Married	10	4.1
	Single	232	95.9
Employment status	Employed	27	11.2
	Unemployed	215	88.8
Place of Residence	Urban	190	78.5
	Rural	52	21.5
Year of Study	2^nd^	81	33.5
	3^rd^	67	27.7
	4^th^	42	17.4
	5^th^	52	21.5
Gadgets Used	Computer	12	5.0
	Laptop PC	13	5.4
	Smartphone	210	86.8
	Tablet	7	2.9
Source of Internet	Mobile data	205	84.7
	Wi-Fi Wireless	37	15.3
Ever participated in E-Learning?	Yes	235	97.1
	No	7	2.9

### Comparison of the effectiveness of online learning against traditional face-to-face

**Fig. 1:**
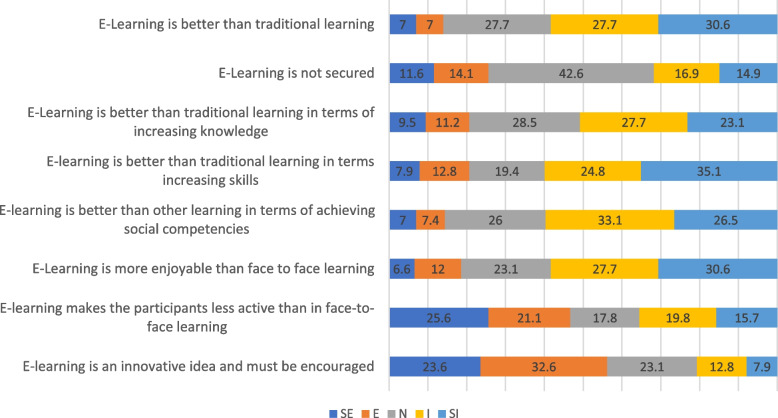
Comparison of the effectiveness of E-learning against traditional face-to-face (*N* = 242). SE = (Strongly Effective) E = (Effective) N = (Neutral) I = (Ineffective SI = (Strongly Ineffective)

Figure 1 illustrates the comparison between traditional learning and online learning. 141 (58%) the of respondents attested that online learning was not better than traditional learning. The largest percentage (42.6%) of respondents were neutral in their beliefs as to whether online learning was secure. 123 (50.8%) respondents disagreed that online learning was better than traditional learning for increasing knowledge, and the majority 145 (59.9%) of the participants agreed that it was not better for increasing skills. Similarly, 144 (59.6%) felt that online learning did not help achieve social competencies more than other learning styles, and 141 (58.3%) felt online learning was not more enjoyable than face-to-face learning. 113 (46.7%) respondents agreed that it made them less active than in face-to-face learning. However, the majority 136 (56.2%) of the respondent supported online learning, saying it is innovative and must be encouraged.

### Students' attitude on the perceived usefulness of online learning amid COVID-19

Table 2 shows the attitude toward the perceived usefulness of online learning among the respondents. It was found that most of the respondents disagreed that online learning could solve many educational problems, improve their access to other learning material, help them achieve better results, increase learners’ engagement in learning, improve teacher and student interaction ( 46.3%,52.0%,42.6%,63.3 and 67.8% respectively). More than half, 176 (72.7%) of the respondents, said that it helped them in saving time and 109 (45.1%) felt that the University should adopt online learning for the students. 2.9 (SD: ± 0.5) was the overall mean score for perceived usefulness.

**Table 2:** Students' attitude on the perceived usefulness of online learning amid COVID-19

Statement	SD	DA	N	A	SA	Mean ± SD
**Perceived usefulness**						**2.9 ± 0.5**
E-learning can solve many of the educational problems	52(21.5)	60 (24.8)	50(20.7)	53(21.9)	27 (11.1)	2.8 ± 1.3
E-learning saves time	11 (4.6)	19 (7.9)	36(14.9)	113(46.)	63 (26.0)	3.4 ± 1.2
E-learning improves access to learning material	20 (8.3)	41 (16.9)	55(22.7)	71(29.3)	55 (22.7)	2.9 ± 1.3
E-learning helps me to achieve better results	42(17.4)	61 (25.2)	60(24.8)	41(16.9)	38 (15.7)	2.3 ± 1.2
E-learning increases learner’s engagement in learning	66(27.3)	87 (36.0)	49(20.3)	25(10.3)	15 (6.2)	2.2 ± 1.2
E- learning improves teacher and students’ interaction	81(33.5)	83 (34.3)	41(16.9)	22 (9.1)	15 (6.2)	2.4 ± 1.1
E-learning increases my understanding of concept	56(23.1)	92 (38.0)	62(25.6)	16 (6.6)	16 (6.6)	2.9 ± 1.2
E-learning has created more problems than it solved	27(11.2)	63 (26.0)	79(32.6)	45(18.6)	28 (11.6)	2.3 ± 1.1
E-learning is too time consuming to use	55(22.7)	100(41.3)	57(23.6)	19 (7.9)	11 (4.6)	2.8 ± 1.2
E-learning has had little impact on me	34(14.1)	70 (28.9)	66(27.3)	44(18.2)	28 (11.6)	2.7 ± 1.1
E-learning is as informative as the teacher	37(15.3)	64 (26.5)	79(32.6)	48(19.8)	14 (5.8)	3.2 ± 1.2
E-learning will never replace other forms of teaching and learning	16 (6.6)	50 (20.7)	85(35.1)	45(18.6)	46 (19.0)	3.0 ± 1.1
E-learning help to reinforce my knowledge	21 (8.7)	61 (25.2)	83(34.3)	56(23.1)	21 (8.7)	3.0 ± 1.2
E-learning helps me to organize my work	22 (9.1)	70 (28.9)	70(28.9)	49(20.3)	31 (12.8)	3.4 ± 1.3
E-learning helps me to catch up missed lectures	24 (9.9)	39 (16.1)	43(17.8)	82(33.9)	54 (22.3)	3.4 ± 1.2
E-learning increases my effectiveness to create presentations	19 (7.9)	39 (16.1)	57(23.6)	81(33.5)	46 (19.0)	3.4 ± 1.2
E-learning increases my research capability	22 (9.1)	29 (12.0)	64(26.5)	81(33.5)	46 (19.0)	3.4 ± 1.2
Universities should adapt E-learning for their students	47(19.4)	27 (11.2)	59(24.4)	67(27.7)	42 (17.4)	3.1 ± 1.4

*SD* Strongly disagree, *DA* Disagree, *N* Neutral, *A* Agree, *SA* Strongly agree

### Students’ attitude on the intention to adapt to online learning during Covid-19

Table 3 represents the students’ intention to adapt to online learning. The majority 137 (56.6%) of the respondents, disagreed that online learning made them uncomfortable, and 122 (50.4%) disagreed with the statement that it was a way of dehumanising the learning process. More than half 142 (58.6%) of respondents agreed to participate in future online learning courses, and 95 (39.2%) also agreed to make plans to buy a computer. 2.8 (SD: ± 0.5) was the overall mean score for the intention to adapt to online learning.

**Table 3:** Student’s attitude on the intention to adapt towards E-learning during Covid-19 (*n* = 242)

**Statements**	**SD**	**DA**	**N**	**A**	**SA**	Mean ± SD
**Intention to adapt towards online learning**						**2.8 ± 0.5**
E-learning makes me uncomfortable because I don’t understand it	48(19.8)	89(36.8)	66(27.3)	28(11.6)	11 (4.6)	2.4 ± 1.1
E-learning is a de-humanizing process of learning	40(16.5)	82(33.9)	64(26.5)	34(14.1)	22 (9.1)	2.7 ± 1.2
I dislike the idea of using E-learning	41(16.9)	71(29.3)	57(23.6)	48(19.8)	25(10.3)	2.8 ± 1.2
I am not in favor of E-learning as it leads to social isolation	22 (9.1)	66(27.3)	53(21.9)	64(26.3)	37(15.3)	3.1 ± 1.2
E-learning doesn’t interest me	36(14.9)	75(31.0)	70(28.9)	40(16.5)	21 (8.7)	2.7 ± 1.2
I plan to participate in future E-learning courses	16 (6.6)	28(11.6)	56(23.1)	94(38.8)	48(19.8)	3.5 ± 1.1
I am planning to buy a computer to be able to follow lectures notes online	22 (9.1)	58(24.0)	67(27.7)	62(25.6)	33(13.6)	3.1 ± 1.2
Using E-learning makes learning fun	32(13.2)	68(28.1)	74(30.6)	49(20.3)	19 (7.9)	2.8 ± 1.1
I don’t know what I would do without E-learning	58(24.0)	77(31.8)	71(29.3)	22 (9.1)	14 (5.8)	2.4 ± 1.1

*SD* Strongly disagree, *DA* Disagree, *N* Neutral, *A* Agree, *SA* Strongly agree

### Students’ attitude on ease use of online learning

In this study (Table 4), 156 (64.4%) of the respondents disagreed that online learning was more difficult than using a library. The majority 171 (70.7%) of respondents said they could easily use the notes through the web. Furthermore, 113 (46.7%) of the responded disagreed that internet use was slowing them down, as did 99 (40.9%) who disagreed that technology would eventually enslave them. 2.5 (SD: ± 0.7) was the overall mean score for ease of online learning.

**Table 4:** Student’s attitude on ease of E-learning (*n* = 242)

Statements	SD	DA	N	A	SA	Mean ± SD
**Easy use of online learning**						**2.5 ± 0.7**
Using E-learning is more difficult than using the library	56(23.1)	100(41.3)	39(16.1)	36 (14.9)	11 (4.6)	2.4 ± 1.1
I can’t read the lectures notes through the web	45(18.6)	126(52.1)	39(16.1)	27 (11.2)	5 (2.1)	1.6 ± 1.4
I can’t learn courses through the web	47(19.4)	112(46.3)	55(22.7)	25 (10.3)	3 (1.2)	2.3 ± 0.9
It is difficult to acquire any significant information by using internet	72(29.8)	106(43.8)	34(14.1)	21 (8.7)	9 (3.7)	2.1 ± 1.1
It is difficult to express my thoughts by writing through E- learning	35(14.5)	77 (31.8)	40(16.5)	65 (26.9)	25(10.3)	2.9 ± 1.3
I find that using the internet make me slow	39(16.1)	74 (30.6)	59(24.4)	49 (20.3)	21 (8.7)	2.7 ± 1.2
I feel we are becoming slaves to technology	29(12.0)	70 (28.9)	54(22.3)	52 (21.5)	37(15.3)	3.0 ± 1.3
My interaction with E-learning is not understandable	22 (9.1)	85 (35.1)	84(34.7)	33 (13.6)	18 (7.4)	2.8 ± 1.0

*SD* Strongly disagree, *DA* Disagree, *N* Neutral, *A* Agree, *SA* Strongly agree

### Technical assistance towards online learning

The technical assistance given by the respondents’ institutions concerning the introduction and use of online learning is shown in Table 5. 103 (42.6%) of the respondents agreed that their institution had an updated website, while 105 (43.4%) agreed that the institution should provide support for an online learning training program. In response to whether the institute has sufficient technology for online learning, 123 (50.8%) of the respondents disagreed. 37.2% (90) of respondents did not seek assistance from the university support services. 2.9 (SD: ± 0.9) was the overall mean score for technical assistance towards online learning.

**Table 5:** Technical assistance towards online learning (*n* = 242)

Statements	SD	DA	N	A	SA	Mean ± SD
**Technical assistance**						**2.9 ± 0.9**
My institute has an updated website	38(15.7)	43(17.8)	58(24.0)	76(31.4)	27(11.2)	3.0 ± 1.2
My institute facilitates E-learning training program	27(11.2)	61(25.2)	49(20.3)	83(34.3)	22 (9.1)	3.0 ± 1.2
My institute has adequate technology for E-learning	46(19.0)	77(31.8)	43(17.8)	57(23.6)	19 (7.9)	2.7 ± 1.2
I seek technical assistance from university support services	30(12.4)	60(24.8)	69(28.5)	60(24.8)	23 (9.5)	2.9 ± 1.2

*SD* Strongly disagree, *DA* Disagree, *N* Neutral, *A* Agree, *SA* Strongly agree

### Learning stressors while using online learning

The stressors that the respondents experienced while using online learning are shown in Table 6. The ability to use online learning anxiously was not a concern for 116 (47.9%) of the respondents. The majority, 191(79%) of the participants, agreed that having a slow internet connection made them more stressed, and 89 (36.7%) said that their teachers pressured them to use online learning. 2.9 (SD: ± 0.9) was the overall mean score for learning stressors while using online learning.

**Table 6:** Learning stressor while using online learning (*n* = 242)

Statements	SD	DA	N	A	SA	Mean ± SD
**Learning stressor**						**2.9 ± 0.9**
Feel anxious about my ability to use online learning effectively	39(16.1)	77(31.8)	65(26.9)	42(17.4)	19 (7.9)	3.1 ± 1.1
Slow internet connections stress me	7 (3.0)	22 (9.1)	22 (9.1)	72(29.8)	119(49.2)	2.4 ± 1.2
I feel pressured by my teachers to use online learning for my research/ learning activities	18 (7.4)	75(31.0)	60(24.8)	56(23.1)	33 (13.6)	3.0 ± 1.2

*SD* Strongly disagree, *DA* Disagree, *N* Neutral, *A* Agree, *SA* Strongly agree

### Distant use of online learning

The importance of using online learning from a distance is illustrated in Table 7. The idea of using online learning to reach students who live in remote locations was disagreed by about 118 (48.8%) of respondents, and 159 (65.7%) of respondents agreed that online learning lessens the stress associated with travel. One hundred and fifty-three (63.2%) of the respondents agreed that online learning should be modified to enable married students to combine work and family obligations. Further, 173 (71.5%) of respondents agreed that online learning should be adapted to enable working students to study from home. 3.5 (SD: ± 0.9) was the overall mean score for distance use of online learning.

**Table 7:** Distant use of online learning (*n* = 242)

Statements	SD	DA	N	A	SA	Mean ± SD
**Distant use of E-learning**						**3.5 ± 0.9**
E-learning should be offered fully online to reach students living in remote areas	59(24.4)	59(24.4)	42(17.4)	55 (22.7)	27(11.2)	2.7 ± 1.3
E-learning should be used to reduce travel related stress	14 (5.8)	21 (8.7)	48(19.8)	106(43.8)	53(21.9)	3.6 ± 1.2
E-learning should be adapted to allow married students to balance family and Study demands	7 (2.9)	27(11.2)	55(22.7)	99 (40.9)	54(22.3)	3.7 ± 1.0
E-learning should be adapted to allow working students to study from home	10 (4.1)	17(7.0)	42(17.4)	105(43.4)	68(28.1)	3.8 ± 1.0

*SD* Strongly disagree, *DA* Disagree, *N* Neutral, *A* Agree, *SA* Strongly agree

### Overall attitude of the respondents about online learning

**Fig. 2:**
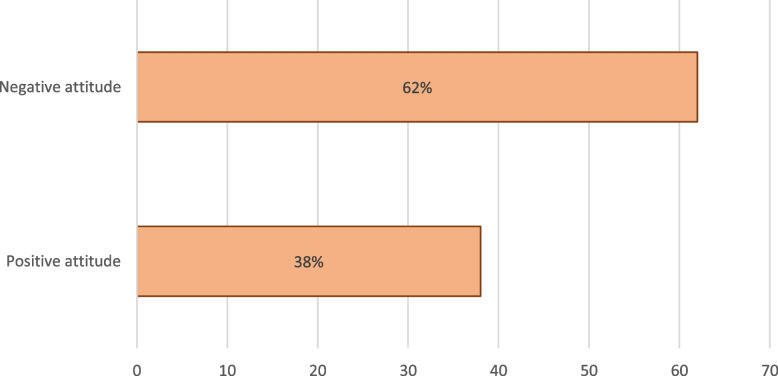
Overall attitude of the respondents about online learning (*n* = 242)

Figure 2 shows the overall attitude of the respondents toward online learning. The overall score was 230, with a mean score of 132.5 (SD: ± 16.3). The population of respondents was divided into two groups: those who had a negative attitude toward online learning (mean score of < 132.5) and those who had a positive attitude (mean score of ≥ 132.5). Therefore, 150 (62%) and 92 (38%) students had overall negative and positive attitudes about online learning, respectively.

### Selected socio-demographic variables associated with attitude regarding online learning.

Table 8 demonstrates that no socio-demographic variables in both bivariate and multivariate logistic regression analysis were predictors of attitude regarding online learning.

**Table 8:** Unadjusted and adjusted logistic regression of factor associated with attitude regarding online learning

Variable	Category	Unadjusted			Adjusted		
		COR	95% CI	*P*-value	AOR	95% CI	*P*-value
Gender	Female	1					
	Male	1.1	0.66 -1.89	0.681	-	-	-
Age	15–19	1					
	20–24	0.7	0.13–4.76	0.783	-	-	-
	25–29	1.7	0.26–11.5	0.567	-	-	-
	< 30	1.1	0.14–9.00	0.912	-	-	-
Marital status	Married	1					
	Single	0.4	0.11–1.43	0.156**	0.4	0.10–0.10	0.137
Employment status	Employed	1					
	Unemployed	0.9	0.39–1.99	0.757	-	-	-
Place of Residence	Urban	1					
	Rural	1.1	0.61–2.13	0.691	-	-	-
Year of Study	2^nd^	1					
	3^rd^	0.7	0.35–1.34	2.71	-	-	-
	4^th^	0.8	0.38–1.73	0.585	-	-	-
	5^th^	0.7	0.34–1.43	0.324	-	-	-
Source of Internet	Mobile data	1					
	Wi-Fi	2.4	0.52–11.0	0.260	2.5	0.54–11.5	0.355
	Wireless	2.1	0.68–6.47	0.197**	2.2	0.71–6.80	0.172

1: Reference variable, *OR* Crude odds ratio, *AOR* Adjusted odds ratio, *CI* Confidence interval

^**^: *P*-value < 0.2 in the unadjusted model

### Barriers towards online learning during the COVID-19 pandemic

**Fig. 3:**
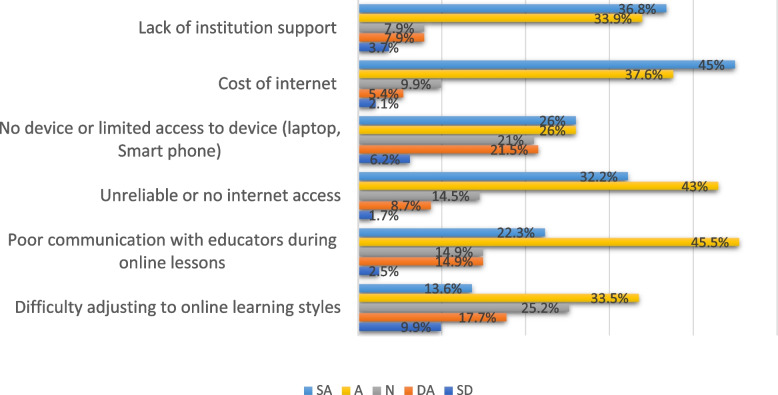
Barriers towards E-learning during the COVID-19 pandemic (*n* = 242). SD: Strongly disagree, DA: Disagree, N: Neutral, A: Agree, SA: Strongly agree

Figure 3 represents barriers to online learning during the Covid -19 pandemic. Around 114 (47.1%) participants agreed they had difficulties adjusting to online learning styles. The majority 171 (70.7%) agreed that the institution did not render them support, and 200 (82.6%) also agreed that the internet is too costly. 182 (75.2%) of respondents agreed that there was unreliable or no internet access. Another 125 (51.6%) of respondents did not own a device and had limited access to a device (laptop, smartphone). Other respondents, 164 (67.8%), revealed that there was usually poor communication with the educators during online lessons.

## Discussion

During the COVID-19 epidemic, nearly all educational institutions used distance learning globally, including Zambia [19–21]. The effectiveness of the distance learning process depends on several factors, including the speed and quality of the internet, the accessibility of online resources, the availability of adequate infrastructure in academic institutions, and the readiness of both teachers and students to adopt this technology [22–24]. This study, therefore, explored pharmacy students’ attitudes toward online learning based on their experience with online learning activities during the pandemic.

Due to their flexibility and ease of carrying, this study found that smartphones became popular online learning gadgets compared to laptops and computers amid the pandemic. Our finding is similar to findings of a study conducted in India [17]. In addition, a study conducted in Ghana found that smartphones are popular because learning can take place anywhere and anytime [25]. Most of the respondents in the present study used mobile data (84.7%) as a source of the internet rather than Wi-Fi, a situation experienced among students in India [26]. Conversely, a study in Nepal reported that most of the students used Wi-Fi for their online learning [27]. We argue that while smartphones are popular and flexible in terms of use, their cost could have limited students from poor backgrounds to effectively participate in online learning. In addition, the high cost of internet connectivity remains a significant issue confronting online learning in countries such as Zambia [28].

### The effectiveness of online learning against traditional face-to-face

Our study revealed that more than half (58.3%) of respondents found online learning less effective than traditional face-to-face learning. Additionally, 50.8% and 59.9% of the respondents felt that traditional face-to-face learning increased knowledge and skills more than online learning respectively. This study's findings are comparable with those of studies conducted in Nepal and India [29, 30]. The reason could be that the students are more used to traditional learning styles and that pharmacy requires task completion through practical application. Students were more satisfied with lectures than with online learning, according to another review that evaluated the use of online learning tools in nursing schools [31]. According to a Taiwanese study, face-to-face instruction was deemed superior to online learning regarding all students' social interaction, contentment, and presence among the lecturers and students [32]. In contrast to the finding of our study, a study showed that online learning is equivalent to traditional learning in terms of academic context [33]. Other studies conducted on students in India favoured using a mixture of face-to-face and online learning [30]. We also argue that the favor for traditional face-to-face learning was because Covid-19 led to an abrupt introduction of online learning, and it was difficult for students and lecturers to adapt easily. Further, institutions of learning were also caught off guard. Despite the preference of face-to-face learning over online learning, most of the respondent in the current supported the use of online learning education, arguing that it is novel and must be promoted.

### Students’ attitude toward the perceived usefulness of online learning amid the Covid-19

According to this study, the majority (72.7%) of the respondents had a negative perception of the usefulness of online learning, and 46.3% of them stated that it could not solve many educational problems, even though the majority (97.1%) of them participated in online learning. Similar findings as the preceding were reported in Pakistan [34]. However, this is in contrast to the findings of other studies, which found positive perceptions of the usefulness of online learning [17, 35]. The main finding of one study conducted in Sri Lanka revealed that attitudes towards online learning are determined by perceived usefulness and perceived student participation [36]. Although the majority had a negative perception of the usefulness of online learning in this study, most acknowledged that it helped them save time. This finding is similar to the finding of the study done in Nepal [17]. It is surprising, however, that one study done in China showed that perceived usefulness does not determine the intention to use an online learning system. Therefore, there is a need for future studies to explore more factors that influence the use of online learning.

### Students’ attitude toward the intention to adapt to online learning during Covid-19

Regarding the intention to adapt online learning, 45.9% of respondents disputed that they were not interested in using online learning, and 58.6% of the students expressed a desire to modify and adapt online learning. This is lower than what was reported in Albania, Nepal, and Pakistan, where 52.5%, 85%, and 100% of students, respectively, wished to adapt to online learning in the near future [17, 35, 37]. The lack of resources to support online learning infrastructure and the high negative attitude noted in the current study, could have influenced the low intention to adapt to online learning in this study. The student's ability to adjust to the online learning approach, depends on their level of awareness, familiarity with information technology, and willingness to participate [38]. Therefore, increasing awareness of the use of technology must be the focus of higher Education to improve adaptability to online learning.

### Students' perceptions of the ease with which they can use online learning

Concerning the ease of use of online learning, the majority of respondents provided satisfactory answers that it was easy to learn online. When asked whether utilising an online course was more challenging than using a library, 64.4% of respondents in this survey disagreed. This is considerably higher than what was reported in Nepal, where 45.5% of respondents found utilising online learning to be simpler than using a library [17]. In the current study, 40.9% of the respondents disagreed that technology would eventually enslave them. This is in line with the study, which also revealed that 38.8% of the respondents dispelled the assertion that technology would eventually enslave them. This is encouraging considering that the majority of the participants in our study were willing to adopt the technology of online learning.

### Technical assistance toward online learning

Scholarship reveals that providing online learning training support is critical for students to implement online learning successfully [39]. In our study, 43.4% of respondents indicated that the institution supported a training program for online learning. This contrasts with what was reported in Nepal, where most respondents had a neutral attitude toward technical assistance [18]. In the current study, 58% of respondents disagreed when asked whether the institute had enough technology for online learning. According to a study conducted in Australia, access to technology was one of the challenges higher education institutions faced when switching to online learning [40]. Therefore, upgrading and having enough technology to support online learning at UNZA is essential to ensuring its success.

### Learning stressors while using online learning

In this study, 48% of the respondents said they were not anxiously worried about their capacity to use online learning. However, 79% of respondents claimed that a slow internet connection contributed to increased stress, and 36.7% said that their teachers forced them to use online learning. This finding is in line with what was observed in Nepal [17]. A good number of studies have shown that the use of distance learning, remote teaching, and the general social turmoil during COVID-19 negatively impacted adolescents' mental health [41, 42]. This study’s findings offer the university authorities a meaningful opportunity to improve internet services to lessen stress and other psychological problems during online learning. Instead of forcing students to use online learning, institutions should review the mechanisms, methods, and practices used to deliver their online courses and programs.

### Distant use of online learning

The usage of social media and distance learning for communication and education has increased because of COVID-19 [43]. In the current study, 49% of respondents disagreed with using online learning to reach students living in rural areas. This could be attributed to the high preference for face-to-face learning noted in this study. In contrast, a study found that 68.3% of respondents supported using online learning to reach students who live in remote areas [17]. Both achievement and learning are enhanced by distance education. The ability to learn at any time and from any location makes distance learning advantageous. For students, staying at home during pandemics is safer and less stressful [44]. However, we argue that, while there was a little option given the situation, online learning in most remote areas of some countries, such as Zambia, can be constrained by poor internet access and constant power outages or completely lack of electricity facilities.

### The overall attitude of respondents regarding online learning

Overall, only 38% of the students in this study had a positive attitude toward online learning. This is much lower than what was reported (43% in Iran, 54.1% in Nepal, and 77% in Pakistan) [17, 34, 45, 46]. Other studies in India and Jordan reported 30.8% and 26.8% positive attitudes toward e-learning, respectively [30, 47]. The lower levels of positive attitude towards online learning in our study can be attributed to poor internet connectivity and a lack of efficient devices used by students during online learning. Therefore, the UNZA should improve the quality of online Education and implement clear guidelines if student behavior towards online learning needs to be improved.

### Factors associated with an attitude toward online learning

The literature reviewed shows that factors associated with an attitude towards online learning vary according to the institution and country. In this study, no socio-demographic characteristics (age, employment status, residence, year of study, and internet source) significantly influenced respondents’ attitudes about online learning. This is supported by the findings of other studies conducted in West Bengal and Nepal [18, 47]. One study discovered that male students preferred online learning to female students. Another study found that male students significantly favoured online learning than female students [48]. Gender, place of residence, level of education, and previous experience were found by one study to have a relationship with students’ attitude toward and online learning [49].

### Barriers to online learning

The most frequently cited barriers to online learning by respondents in our study were the cost of the internet, a lack of institutional support, unreliable or no internet, and limited access to devices (laptops and smartphones). This finding is supported by research that identified unreliable internet as one of the major barriers to online learning [48-50]. Other studies cited the cost of the internet and lack of access to laptops and smartphones as the most frequent barriers to e-learning [49, 51]. It can be suggested that investment in affordable internet services is one way of easing the challenges associated with online learning, in addition to the provision of soft loan schemes for gadgets such as laptops and smartphones for students.

### Limitations of the study

Conducting this study in a single discipline with a specific target group (“the pharmacy students”) is considered a study limitation that could hinder the generalisation of the findings.

## Conclusion

In this study, students at the University of Zambia Ridgeway Campus preferred traditional face-to-face learning over online learning, as evidenced by the overall 62% negative attitude exhibited by the respondent. However, most students supported online learning, saying it is an innovative idea that must be encouraged. In this study, the major barriers to online learning were high internet costs, a lack of institutional support, and unreliable or no internet. Students consider online learning to be beneficial, although not entirely effective, amid a pandemic. It can be concluded that the institution should invest in advanced technologies and good internet infrastructure to make pharmacy lectures enjoyable for the students. Additionally, students and lecturers should be well-trained in using advanced online learning technologies for pharmacy lessons. From the findings, it is evident that online learning evoked both positive and negative adaptive attitudes and perceptions among students. Therefore, this presents an opportunity for educational institutions to integrate the positive attributes of both online and traditional face-to-face learning and advocate for hybridisation in the delivery of course content in universities. We argue that online learning is the new normal in the education sector, and therefore governments and institutions should invest in ICT’s soft and hard infrastructure.

## Data Availability

The datasets used and/or analysed during the current study are available from the corresponding author on reasonable request.
